# Stress-Induced Evolution of the Nucleolus: The Role of Ribosomal Intergenic Spacer (rIGS) Transcripts

**DOI:** 10.3390/biom14101333

**Published:** 2024-10-20

**Authors:** Anastasia A. Gavrilova, Margarita V. Neklesova, Yuliya A. Zagryadskaya, Irina M. Kuznetsova, Konstantin K. Turoverov, Alexander V. Fonin

**Affiliations:** 1 Laboratory of Structural Dynamics, Stability and Folding of Proteins, Institute of Cytology, Russian Academy of Sciences, St. Petersburg 194064, Russia; asultanbekova@incras.ru (A.A.G.); neklesova.m@mail.ru (M.V.N.); imk@incras.ru (I.M.K.); kkt@incras.ru (K.K.T.); 2 Moscow Center for Advanced Studies, Moscow 123592, Russia; 1989july@mail.ru

**Keywords:** LLPS, non-coding RNA, intrinsically disordered proteins, membrane-less organelles, stress response, nucleolus

## Abstract

It became clear more than 20 years ago that the nucleolus not only performs the most important biological function of assembling ribonucleic particles but is also a key controller of many cellular processes, participating in cellular adaptation to stress. The nucleolus’s multifunctionality is due to the peculiarities of its biogenesis. The nucleolus is a multilayered biomolecular condensate formed by liquid–liquid phase separation (LLPS). In this review, we focus on changes occurring in the nucleolus during cellular stress, molecular features of the nucleolar response to abnormal and stressful conditions, and the role of long non-coding RNAs transcribed from the intergenic spacer region of ribosomal DNA (IGS rDNA).

## 1. Introduction

The discovery of biomolecular condensates has become a new stage in the development of studies of cell response to stress. Most compartments formed or rearranged in response to stress have liquid-like properties. For example, Cajal bodies [1], paraspeckles [2], nuclear speckles [3], PML nuclear bodies [4], and P-bodies [5] reorganize in response to various types of stress. NELF bodies [6], nucleolar stress bodies [7], and stress granules [8] assemble in response to stress. The transition of these condensates from a liquid to a solid state is associated with human pathologies and diseases [9]. Stresses such as heat and osmotic shock lead to protein misfolding, which is common in type II diabetes, Alzheimer’s, Huntington’s, and Parkinson’s diseases, while oxidative stress can cause cardiovascular diseases, Parkinson’s disease and cancer [10,11,12].

Nucleoli are membrane-less organelles (MLOs) and are dynamic structures with highly mobile components that can diffuse in and out of the nucleoplasm. The main role in the formation of the nucleolus structure, as well as the nucleolar response to stress, is played by liquid–liquid phase separation (LLPS), which causes the self-organization of cellular compartments. The nucleolus was first discovered by Felice Fontana in eel cells in 1774. In 1781, Fontana described the nucleolus after finding it in the slime from an eel’s skin. Theodor Schwann, in his 1839 treatise “Microscopic researches into the accordance in the structure and growth of animals and plants” [13], mentions a small body identified by Matthias Jakob Schleiden that looks like a thick ring or a thick-walled hollow globule. He calls this structure “Kernkörperchen”. In the English translation of the work in 1947, the structure was called “nucleolus”. Little was known about the nucleolus until 1964, when Donald D. Brown and J. B. Gurdon discovered in Xenopus laevis that anucleolate embryos were unable to synthesize rRNA and stopped developing when the maternal supply of ribosomes was exhausted [14]. It was later shown that the DNA in the nucleolus encoded ribosomal RNA, and the role of the nucleolus as a site of ribosome synthesis was established [15,16,17]. Further study revealed that the nucleolus is involved in many cellular processes, such as oncogenesis, DNA repair, and cell cycle progression and aging [18,19,20].

It is well recognized that human diseases such as cancer, viral infections, cardiovascular and neurological disorders are linked to disruptions in the structure and function of the nucleolus [21]. The nucleolus’s function as a stress signaling center is primarily responsible for its involvement in the genesis of illness [22]. In this review, we will consider the function of non-coding RNA in regulating the nucleolar stress response.

## 2. Evolution of Nucleolar Structures

### 2.1. Nucleolus

The nucleolus is the largest substructure of the nucleus, a multiphase fluid condensate formed around the nucleolar organizer regions (NORs) [23]. NORs are tandem arrays of ribosomal gene repeats of 10 acrocentric chromosomes (chromosome pairs 13, 14, 15, 21, 22). Nucleolus assembly occurs over a relatively long period. In HeLa cells, the cell cycle lasts 22 h; complete assembly of the organelle takes about 2 h, while disassembly requires 30 min [24]. It is believed that nucleolus formation may be initiated by complex coacervation of pre-rRNA molecules and a number of intrinsically disordered nucleolar proteins, such as nucleolin, fibrillarin, and nucleophosmin [25,26]. The components of the nucleolus are ribosomal DNA (rDNA), pre-ribosomal RNA (pre-rRNA), ribosomal RNA (rRNA), transcription factors, and RNA- and DNA-binding proteins [27]. The existence of the nucleolus is determined by the expression of rDNA. In the cell cycle of higher eukaryotes, rDNA expression begins at the end of telophase and is blocked during mitosis. During early prophase, DNA polymerase I activity decreases by about 30% during early prophase and stops in late prophase due to sequential phosphorylation of its cofactors, the CDK1/cyclin B [26]. Chromatin condenses, and a perichromosomal compartment forms, consisting of condensed chromosomes and a perichromosomal layer containing pre-rRNA, U3 snoRNA, and several extant ribosomal proteins. While rDNA genes switch numerous components, including UBF and most RNA polymerase I subunits, to an inactive form, other proteins transiently dissociate from transcriptional complexes and relocate to the cytoplasm. After transcription arrest, pre-RNA, a factor in the nucleolus, gradually diffuses into the cytoplasm, and a coat is maintained around the condensed chromosomes in metaphase. They are also found in a few foci called nucleolar-derived foci (NDFs), which are converted into cytosolic daughter cells before early G1. At the end of prophase, final chromosome condensation and nuclear lineage disruption occur, and the nucleolus disappears [27]. It has been shown that although pre-rRNA transcription ceases during prophase, the transcription machinery remains associated with rDNA in the NOR. The presence of transcription factors DNA polymerase I, SL1, TTF1, and UBF in the NOR during mitosis has been demonstrated by electron microscopy [28]. During telophase, as mitotic exit proceeds, the transcription machinery is reactivated in the NOR [20], but this event is not necessary for nucleolus formation. As chromosomes begin to unwind, the perichromosomal envelope disintegrates into prenucleolar bodies (PNBs), which contain pre-rRNA but not RNA polymerase I machinery. PNBs are later actively targeted at active NORs, forming postmitotic nucleoli. Individual active NORs begin to form nucleoli; they eventually fuse to form more advanced nucleoli [28].

It was shown [29] that nucleolus formation after mitosis is the result of direct recruitment of processing factors and pre-rRNA to the NORs before or at the beginning of rDNA transcription. As the cell cycle progresses, the numerous nucleoli that form merge to form a larger aggregate [23,30,31]. The multiphase structure of the nucleolus is formed by the following elements: the fibrillar center (FC), the dense fibrillar component (DFC), and the granular component (GC) [32].

The FC is where rRNA gene transcription takes place. Transcriptionally active rDNA genes are located at the FC/DFC border region. rDNA genes form loop structures. This loop formation promotes increased transcription speed because such structure allows a switch from termination to reinitiation of transcription [33].

The DFC contains synthesized pre-rRNA. Processing of the 47S precursor transcript pre-rRNA is carried out in the DFC. Pre-rRNA undergoes either co- or post-transcriptional processing by ribosome assembly factors (AFs) and is modified by small nucleolar ribonucleoproteins (snoRNPs) to form 28S, 18S, and 5.8S rRNA. These snoRNP-mediated modifications include 2’-O-methylation and pseudouridine formation [34,35]. AFs also facilitate the binding of structurally constitutive ribosomal proteins (RPs) and the folding of maturing subunits. Defects in RPs can cause a nucleolar stress response, which at the organismal level can cause a class of rare human diseases called ribosomopathies. The scaffold protein of DFC is probably the 2′-O-methyltransferase fibrillarin, forming a cluster network in the DFC surrounding the FC [36].

Late rRNA processing and assembly of ribosomal units occur in the GC surrounding the FC and DFC. Interacting with RPs, 28S, 18S, and 5.8S rRNAs form small and large pre-ribosome subunits, each of which is exported to the cytoplasm, modified, and involved in the formation of mature 40S and 60S ribosome subunits [37,38].

An important feature of the GC is its function as a non-membrane-bound protein quality control compartment. It is characterized by a chaperone-like ability to temporarily store misfolded proteins, preventing their irreversible aggregation and keeping them competent for refolding by Hsp70 [37].

MicroRNAs are also known to play an important role in regulating ribosome biogenesis. They are a class of non-coding (nc) RNAs approximately 22 nt in length. How microRNAs regulate ribosome biogenesis remains to be elucidated, but several microRNAs have been shown to be involved in RNA pol I, RP transcription, and 60S assembly [38]. AGO2 has also been detected in the nucleolus along with several microRNAs, although their exact biological function there remains unclear [39]. A recent study screened for microRNAs involved in cell cycle regulation, cellular proliferation, and localization within the nucleolus. Several microRNAs have been shown to be strong inhibitors of pre-18S pre-rRNA processing by way of potent downregulation of the levels of the ribosomal protein mRNA. The screening results highlight the important role of microRNAs in the dysregulation of ribosome biogenesis. The extent to which microRNAs can influence ribosome biogenesis and thereby mediate various ribosomopathies remains to be studied [40].

Remarkably, it is not only RNA Pol I and RNA Pol III that influence ribosomal RNA synthesis. The study showed that RNA Pol II in the nucleoli acts in close proximity to genes encoding rRNA and controls its expression [41].

It is unclear how much of the nucleolar structure can be explained by a liquid–liquid phase separation (LLPS) framework. Nucleolar subcompartments can, on the one hand, merge and reform upon bleaching [36]. Furthermore, droplets made in vitro from scaffold proteins derived from various nucleolar components have the ability to subcompartmentalize and form structures [25]. It has been demonstrated that RNA recognition motifs are necessary to preserve phase separation and that droplet formation is driven by the disordered domains of nucleophosmin (NPM) and fibrillarin (fbl), which are important components of DFC and GC [34,42]. However, ribosome maturation takes place in a system that is not in equilibrium, which gives rise to features that are not similar to liquids. The study [38] showed that during short-term treatment with polymerase I inhibitor, the rough outer edges of GC become rounded with a liquid-like appearance and exhibit properties more typical of equilibrium fluids. A decrease in metabolic activity leads to a change in the surface tension at the FC/DFC interface and an increase in FC, migration of this structure to the periphery of the nucleolus. Thus, this structure can be considered as a model of “local equilibrium” in a nonequilibrium system [43].

The nucleolus contains RNA, proteins, and ribonucleoprotein (RNP) assemblies with remarkably high estimated local RNA concentrations (around 50 mg/mL in contrast to 0.5 mg/mL in the nucleoplasm). The protein concentration in the nucleolus is approximately twice that in the nucleoplasm [44]. 

**Figure 1:**
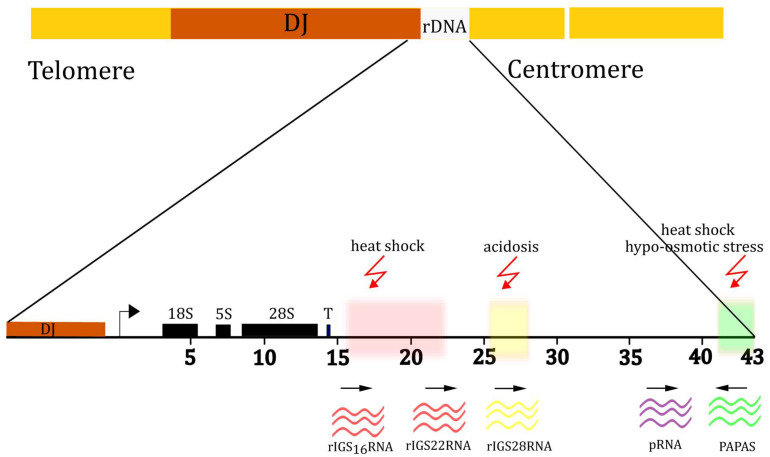
The ribosomal DNA cassette (rDNA cassette). The rDNA cassette contains sequences encoding the pre-RNA and the ribosomal intergenic spacer (rIGS). In humans, the cassette size is approximately 43 kb; they are located on the p-arms of five chromosomes. Distal Junction is a sequence approximately 400 kb long that flanks the ribosomal gene repeat. Polymerase I transcribes several functional noncoding RNAs from rIGS. rIGS 16 RNA and rIGS 22 RNA are synthesized during heat shock, and rIGS 28 RNA is synthesized under acidosis conditions. pRNA is transcribed from spacer promoters upstream of the pre-rRNA transcription start site. PAPAS are a set of antisense RNAs that are synthesized by Pol II. PAPAS transcripts do not have a common promoter; their transcription begins at random sites and can span both pre-rRNA coding and IGS regions longer than 10 kb.

The nucleolus is a compartment with a very low DNA concentration, but in human cells, mature nucleoli are surrounded by perinucleolar heterochromatin (PNH), which includes sequences around the centromeres and, partially, the p-arms of the acrocentric chromosomes. Approximately 400 kb, located distal to the rDNA array, are common to the 5 pairs of chromosomes carrying NOR and are called the Distal Junction (DJ). In mature interphase nucleoli, rDNA sequences extend into the nucleolar interior and are anchored by DJ sequences located in perinucleolar heterochromatin (PNH) [40] (Figure 1). Sequences around the nucleolus also include nucleolus-associated domains (NADs). In mammalian cells, identification of the nucleolus-associated chromatin domain (NAD) revealed that all 23 human chromosomes possess at least one nucleolus-associated region. NADs consist of genomic regions enriched in silent chromatin modifications associated with transcriptional repression, such as DNA methylation and/or histone 3 lysine 9 [45,46].

In addition to coordinating rRNA transcription biogenesis, the nucleolus is involved in the regulation of the cell cycle, mitosis [47], and in the response to some types of stress [31,48,49]. 

### 2.2. Reorganization of the Nucleolus Under Stress

The most important cellular function is maintenance of homeostasis and adaptation to deviations from the normal growth environment by modulating metabolic processes. The adaptive response of the cell is mediated by the activation of various signaling pathways, specifically determined by the type and severity of damage [50].

Disruption of any stage of ribosome biogenesis leads to nucleolar stress. Under normal growth conditions, levels of p53 protein are regulated by the E3 ubiquitin ligase, MDM2. MDM2 ubiquitinates p53, targeting it for degradation by 26S proteasome. When ribosome biogenesis is interrupted by stress, the 5S RNP, composed of RPL5, RPL11, and the 5S rRNA, binds and sequesters MDM2 in the nucleoplasm, stabilizing p53 and leading to p21 induction, cell cycle arrest, and apoptosis [51].

In 1999, after the discovery that the cell cycle regulator phosphatase Cdc14 and MDM2 can be transiently localized to the nucleoli [52], the nucleolar sequestration hypothesis was put forward [53]. A striking example of nucleolar sequestration is the retention of the E3 von Hippel-Lindau ubiquitin ligase (VHL) protein in the nucleolus [54]. It has been shown that increased hydrogen ion concentrations promote pH-dependent nucleolar sequestration of VHL, causing a transient and reversible loss of VHL function. Stress-responsive proteins such as p53, mdm2, and PML are sequestered by the nucleolus in response to stress [55,56,57]. Notably, proteins that undergo nucleolar sequestration are prone to aggregation [48,54,58,59]. This has been demonstrated for Cdc14 [60], MDM2 [48], VHL [58], RNF8 [61], DNMT1 [48], and Piwi [62]. 

**Figure 2:**
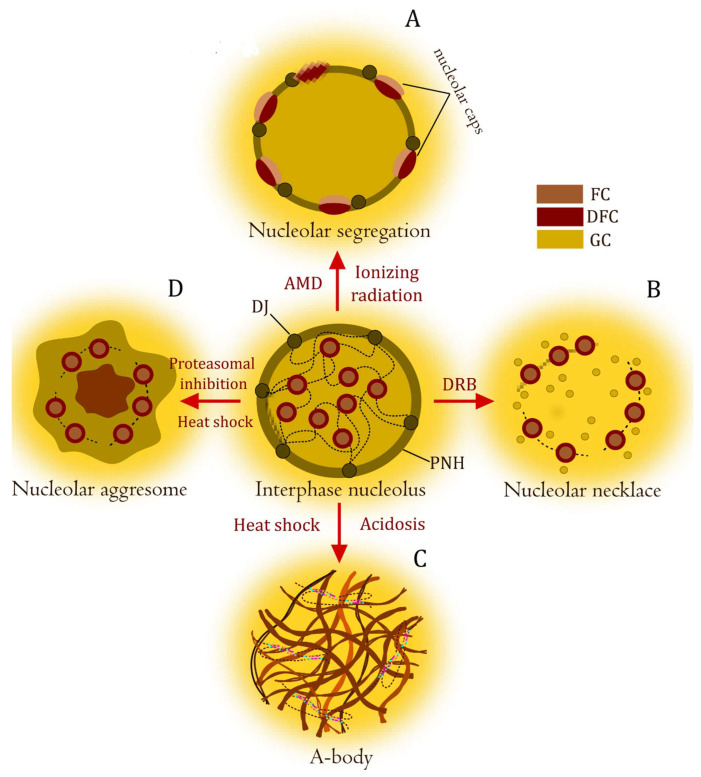
Nucleolar transformation in response to stress. The interphase nucleolus with characteristic tripartite structure is shown in the center. Panels (**A**–**D**) show structures that arise under different stressful influences. (**A**) Nucleolar segregation or nucleolar caps are formed when RNA pol I transcription is inactivated. Actinomycin D or ionizing radiation induces rDNA double-strand breaks (DSBs), resulting in the formation of nucleolar capsules adjacent to their DJ anchor. (**B**) Upon DRB treatment, RNA pol I transcription is active, but rRNA processing is converted to form a nucleolar necklace. (**C**) When exposed to heat shock and acidosis, the nucleolus transforms into an electron-dense fibrillar organization, the A-body. The fibers contain immobilized proteins in an amyloid-like state. (**D**) Nucleolar aggresomes are formed upon proteotoxic insults such as proteasome inhibition and heat shock. This may or may not involve inhibited RNA pol I activity.

Different types of stress induce nucleolar reorganization and the formation of stress-specific intranucleolar structures (Figure 2). Thus, nucleolar caps are formed upon DNA damage by ionizing radiation or inhibition of RNA pol I by actinomycin D. Segregation of the nucleolus and migration of rDNA repeats from individual NORs to nucleolar caps located immediately adjacent to their DJ anchor occur (Figure 2B). Such nucleolar caps contain FC and DFC around GC components [63]. Nucleolar functions are reversibly altered upon treatment of cells with 5,6-dichloro-1-β-D-ribofuranosylbenzimidazole (DRB). DRB reduces speed but does not inhibit rDNA transcription by RNA polymerase I. As a result, the nucleolus is reorganized to form a “nucleolar necklace” (Figure 2C). Each bead of the necklace includes FC and DFC and contains rDNA, rRNA, pol I, DNA topoisomerase I, UBF, and fibrillarin [64]. The formation of such structures under stress has several advantages. First, damaged rDNA exposed on the nucleolar surface becomes accessible to repair factors. Second, the nucleolar caps or beads of the necklace concentrate high levels of homologous sequences in close proximity to each other, which may facilitate repair by homologous recombination. Interestingly, each NOR present in the large fused nucleolus forms its own discrete nucleolar cap in response to DNA damage [65].

Some stress factors lead to the formation of novel nuclear stress bodies [48,49,66]. These compartments are similar but are probably different structures. As a result of inhibition of proteasome activity, aggresome-like structures are formed in the nucleoli. They do not have nucleolar marker proteins, rRNA, and do not synthesize pre-rRNA. Nuclear aggresomes contain polyadenylated RNA, conjugated ubiquitin, and numerous target proteins of nucleoplasmic proteasomes [51] (Figure 2D). In response to heat shock and acidosis, the nucleoli are reorganized into reversible amyloid bodies (A-bodies), which form around the intergenic spacer ribosomal RNA [48,49,67] (Figure 2C). A-bodies are interesting, first of all, because, having a structural similarity to pathological amyloids, they perform important biological functions and can reversibly assemble and disassemble. 

### 2.3. Biogenesis of A-Bodies

The main feature of A-bodies is that they are physiologically reversible amyloids [48,49]. Biogenesis of A-bodies occurs in response to heat shock and low pH of the environment [68]. After normal growth conditions are restored, they are disassembled [49,69]. Currently, there is a model according to which stress exposure mediates transcription from the intergenic spacer (IGS) part of rDNA, rich in dinucleotide repeats. These transcribed regions are located ~16 kb, ~22 kb, and ~28 kb downstream of the rRNA transcription start site. These long non-coding RNAs (lncRNAs) will be discussed in detail below. Certain proteins are sequestered in the nucleolus, and several “foci” are formed. These “foci” are spherical, contain mobile proteins, and can undergo fusion, thereby exhibiting some properties inherent in dynamic condensates. Then these foci mature into aggregates that contain immobilized proteins. Further capture and immobilization of free proteins occurs. Maturation of A-bodies ends as soon as the pools of cellular mobile proteins are depleted, which leads to the emergence of a distinct amyloid organization. Disassembly of A-bodies occurs within 1–2 h after the cessation of the stimulus; this process requires heat shock proteins hsp70 and hsp90 [68,70].

It is believed that proteins recruited to A-bodies have an ACM (amyloid-converting motif), which has a bipartite structure: on one side, an arginine-histidine-rich domain, which, due to its positive charge, interacts with RNA, and on the other side, a motif with a high tendency to form amyloid fibrils (fibrillogenesis). This ensures, on the one hand, interaction with RNA and, on the other, amyloidogenesis. Examples of such proteins are VHL, CDC73, MDM2, POLD1, etc. It has been shown that the proteomic composition of A-bodies formed as a result of exposure to heat shock and low pH overlaps by approximately 20% [71]. In the work [72], two related pairs of proteins were used as model systems to identify critical structural elements that mediate heat shock-specific amyloid aggregation: DDX39A, DDX39B, and hnRNPA0, hnRNPA1. DDX39A and DDX39B have 90% identical sequence and similar shape [73]. However, DDX39B is present only in A-bodies formed as a result of acidotic exposure, while DDX39A is characteristic of both types of A-bodies [72]. In the pair hnRNPA0 and hnRNPA1, both proteins have two N-terminal globular domains and a large C-terminal IDR, but only hnRNPA0 is a putative resident of the A-body during heat shock exposure. The specificity of recruitment was shown to result from local conformational changes induced by temperature changes in distinct structural pockets of these proteins. In both protein pairs, regulation of A-body targeting and protein aggregation was shown to be mediated by globular domains and independent of intrinsically disordered regions. Thus, some proteins possess temperature-sensitive structures and may mediate specific recruitment to A-bodies. However, prior or combined heat shock acidosis was known to prevent sequestration of heat shock-specific FEN1 under heat shock conditions. Clearly, if heat exposure alone were sufficient to refold FEN1, the protein would be recruited to A-bodies [71]. It is possible that acidotic conditions block elements of the heat shock signaling pathway, such as expression of heat shock-inducible transcripts. Accordingly, protein sequestration in A-bodies is suppressed. The mechanism of specific recruitment is probably based not only on the properties of the protein but also on the structure of the primer-noncoding RNA transcribed from the IGS.

Nuclear aggresomes and A-bodies have the following similarities: they (1) are formed in the nucleolus in response to stress; (2) contain proteins that are not characteristic of interphase nucleoli; (3) accumulate RNA that is not characteristic of the nucleolus. Despite the obvious similarities, A-bodies have a unique fibrillar organization characteristic of amyloids, while nucleolar aggresomes have a cavernous shape [68]. In addition, A-bodies are rIGSRNA-dependent, while nucleolar aggresomes contain polyadenylated RNA.

A study by a group led by Serena Carra [74] showed that the products of incomplete translation—defective ribosomal products (DRiPs)—diffuse into the nucleus and accumulate in nucleoli and PML bodies. In this case, both the nucleoli and the PML bodies lose their dynamism and harden. The authors of the article suggested that the organelles are a type of A-bodies. However, it has not yet been shown that these bodies are RNA-dependent.

A recently published study using Full-length-and-mRNA sequencing showed that A-bodies formed as a result of heat shock contain polyadenylated RNA synthesized by TENT4b [75]. This RNA functions as polyanionic stimulators of amyloidogenesis in vivo and in vitro. It is composed of small rRNA fragments linked to long, linear mixed tails. According to the authors, rIGSRNA recruits enzyme TENT4b in intranucleolar foci to coordinate this RNA synthesis, driving their amyloidogenic phase transition. It remains to be seen whether polyadenylated RNA is synthesized in acidosis.

### 2.4. Ribosomal Intergenic Spacer (rIGS)

In humans, ~400 rDNA repeats are distributed among five pairs of NOR regions on the short arms of acrocentric chromosomes [76,77,78], and range in size from 0.05 Mb to >6 Mb [34]. Most repeats are organized as tandem arrays. The number of rDNA repeats in individual human NORs ranges from 1–3 to >140 [79,80,81]. A repeat unit of rDNA encodes a copy of the 18S, 5.8S, and 28S rRNA sequences. These sequences are separated by two internal transcribed spacer (ITS) sequences (ITS1 and ITS2) and flanked by external transcribed spacers (ETS) (3′ ETS and 5′ ETS), which are removed during processing. Repeating units of rRNA are separated by the IGS (Figure 1). Telomere-to-telomere (T2T) sequencing has recently shown that within cells, the 45 kb rDNA repeats are nearly identical but not completely identical. The length of these arrays varies between individuals, with an average of 315 rDNA copies and a standard deviation of 104 copies [38].

In some fungal species, the IGS contains the 5S rRNA gene (Saccharomyces cerevisiae and Flammulina velutipes), but in other fungi, animals, including humans, plants, and protists, 5S rRNA is transcribed separately, often from separate chromosomes [82,83,84]. The IGS contains enhancer sequences, terminator elements, and promoter regions for ncRNAs consisting of tandem repeats (TRs) [85]. Although the presence of promoters in the IGS has long been known, their biological function and the role of IGS transcripts are not well understood [85]. The length of the IGS in eukaryotes ranges from 2 to 30 kb (Homo sapiens about 30 kb) [74,80]. Most species have an IGS several thousand bases long [86]. Due to the lack of conservation, IGS were long considered functionally inactive. Most of the IGS is formed by satellite DNA (satDNA), which is characteristic of various parts of the genome. SatDNAs are the main component of heterochromatin, which is present in the pericentromeric and subtelomeric regions of chromosomes [87,88,89]. SatDNAs can perform important cellular functions, such as closing the ends of chromosomes [90,91]. SatDNA also forms centromeres [89]. Heat shock is known to initiate transcription of lncRNA from pericentromeric HSATIII repeat arrays specific to primates [92]. As a result, nuclear stress bodies (NSBs) are formed—subnuclear non-membrane organelles. In stressed cells, they participate in rapid, transient, and global reprogramming of gene expression through chromatin remodeling and sequestration of transcription and splicing factors. The formation of these organelles is conceptually similar to the formation of A-bodies; however, NSBs are more dynamic and do not have amyloid properties [93]. 

SatDNA may contain tandemly repeated sequences ranging in length from 2–6 base pairs (called microsatellites) to hundreds of base pairs and several thousand base pairs (macrosatellites), although these designations are rather arbitrary [94,95]. The main feature characteristic of this type of DNA is the tandem arrangement of repeating units.

In the study [96], IGS of 12 species, including Homo sapiens, were analyzed. It was shown that in human IGS, the number of direct repeats (DR) is 1429, which is 62.7% of IGS. Tandem repeats of the R1 type with an average length of 758 bp are also present. 

It is important to note that these lengths often vary within and among individuals. For each of the 12 species studied, the analysis showed that the IGS and ETS sections consist mainly of TR and DR, which, as the authors believe, could have arisen as a result of the entry and exit of transposons. The authors of the study [96] suggest that satDNA found in IGS may represent a special type of transposable elements (TE), in which all enzymes required for proliferation are encoded by the host cell. The transposon transcript is inserted into the DNA break, followed by invasion and reverse transcription, which often occur with errors. The host DNA polymerase then synthesizes the opposite strand. Target site duplications, which represent DRs, are formed on both flanks of the insertion during synthesis and integration. Although TEs and satDNA have been largely studied independently, it is now believed that there is a link between these two classes of repeats in the evolution of many eukaryotic genomes. One of the best-studied cases of TE intrusion into satDNA regions is the mammalian non-LTR L1 (LINE-1) elements, which account for 21% of the human genome [97]. Another example is the insertion of R2 retrotransposons into the 28S rRNA genes [98]. It is likely that DRs in IGS are traces of target site duplications of ancient transposition events. The biological role of IGS transcripts is not well understood, but they have been shown to participate in chromatin remodeling [85], modulation of rRNA transcription [99], regulation of splicing [100,101], and stress response. Below, we will discuss in more detail some transcripts involved in cellular adaptation to stress conditions such as heat shock, acidosis, hypotonic stress, or serum starvation.

### 2.5. IGS Transcripts in the Regulation of the Nucleolar Stress Response

In the IGS region, non-coding RNAs are transcribed during stress exposure, triggering various functions [102]. 

**Figure 3:**
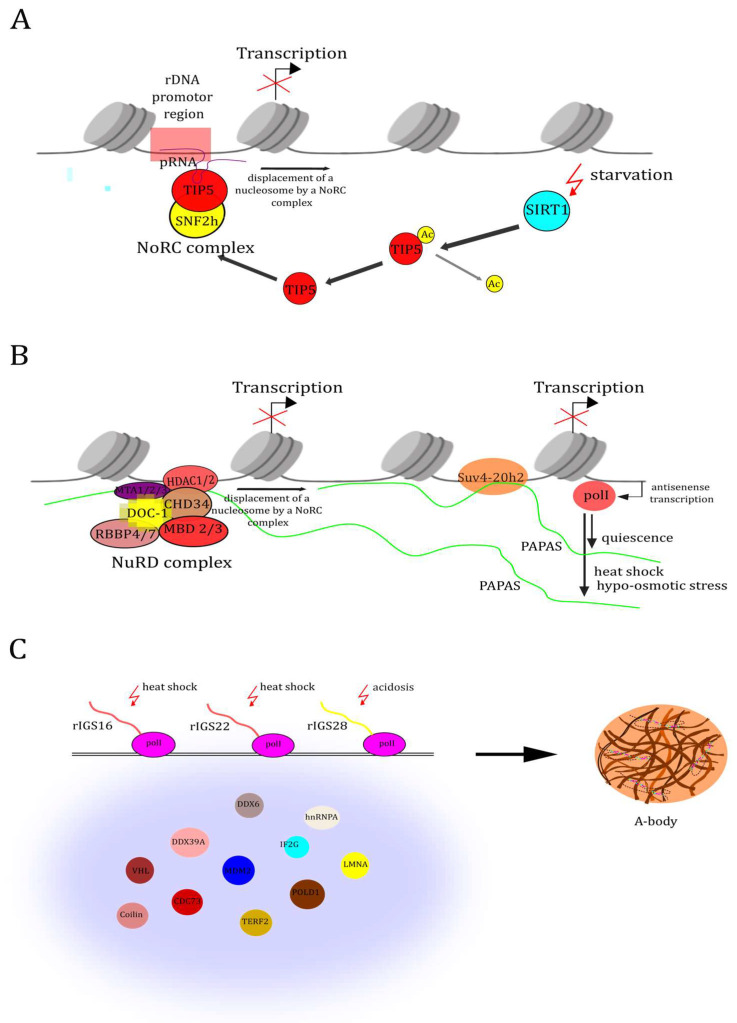
Stress-induced transcription of non-coding RNAs from the IGS region. (**A**) pRNA recruits the Nucleolar Remodeling Complex (NoRC) to the promoter and, due to its hairpin structure, binds to one of the NoRC proteins, TIP5, thereby mediating the nucleolar localization of the entire complex. NoRC moves the nucleosome to a repressive position, preventing transcription initiation. (**B**) During cellular quiescence and starvation, the amount of PAPAS increases. The transcripts recruit the histone methyltransferase Suv4-20h2 to the rDNA locus, which installs the repressive H4K20me3 mark on the rDNA, resulting in immediate suppression of rDNA expression. (**C**) During heat shock and acidosis, rIGS 16 RNA, rIGS 22 RNA, and rIGS 28 RNA are transcribed, respectively. These transcripts likely mediate the nucleolar sequestration of VHL, CDC73, MDM2, POLD1, and many other proteins possessing amyloid-converting motifs. The local concentration of proteins with an amyloidogenic propensity in the foci triggers physiological amyloidogenesis and generates nascent amyloid bodies (A-bodies).

NoRC-associated RNA (pRNA) pRNA is transcribed in the IGS regions from the DR-rich promoter region (Figure 3A). pRNA has a stem-loop secondary structure and is associated with the rDNA promoter due to the formation of an RNA–DNA triplex structure [99,103]. At the same time, pRNA is able to recruit the nucleolar remodeling complex (NoRC) to the promoter [85,99]. pRNA, due to its hairpin structure, binds to one of the NoRC proteins TIP5 [104], thereby mediating the nucleolar localization of the entire complex. NoRC, anchored by pRNA to the rDNA promoter, switches the nucleosome to a repressive position, preventing transcription initiation [105]. The pRNA-TIP5 complex interacts with histone deacetylases and histone methyltransferases, promoting transcriptional repression [106]. In addition, poly(ADP-ribose) polymerase-1 binds to TIP5 via pRNA and represses rRNA transcription [107]. Under normal conditions, the pRNA-NoRC pathway is required to repress a significant proportion of rDNA repeats and, ultimately, to control the number of ribosomes in the cell [108]. Under starvation conditions, rRNA transcription is more intensely repressed. Glucose starvation increases the activity of SIRT1 deacetylase, which deacetylates TIP5 by periodically binding it to pRNA, thereby mediating the repression of rDNA transcription [109]. Thus, stress conditions lead to gene repression through post-translational modification of the chromatin remodeling complex component and lncRNA IGS.

#### 2.5.1. LncRNAs PAPAS (Promoter and Pre-rRNA Antisense)

PAPAS is a collection of antisense RNAs transcribed by pol II from IGS, coding sequences, and the rDNA promoter [110] (Figure 3B). PAPAS transcripts do not share a common promoter; their transcription starts at random sites, and the length of the transcripts ranges from 12 kb to 16 kb [111]. LncRNAs PAPAS, like pRNA, form an RNA–DNA triplex. The 3’-poly(A) tails of these transcripts interact with the poly(T) sequence in the enhancer region of the rRNA gene, which is located at position −275/−336 upstream of the rDNA transcription start site. During cellular quiescence, heat shock, and hypo-osmotic stress, PAPAS levels increase [112,113]. Transcripts recruit histone methyltransferase Suv4-20h2 to the rDNA locus, which installs the repressive mark H4K20me3 on rDNA, which leads to immediate suppression of rDNA expression [114]. Under heat shock and hypoosmotic stress, PAPAS recruit the NuRD complex to the rDNA promoter. Histone deacetylation and nucleosome shift occur. Transcription of rDNA stops [115,116].

The mechanisms of action of pRNA and PAPAS are very similar. Both types of transcripts form a DNA–RNA triplex, recruit nucleosome remodeling complexes, as well as special factors that cause repressive histone modifications at rDNA promoters. Thus, pRNA and PAPAS are sense and antisense analogues.

#### 2.5.2. rIGSRNA

The molecular events underlying nucleolar sequestration remain poorly understood, but it is now known that, in some cases, rIGSRNA is involved in this process [48,49,67,116]. In the study [48], it was shown that acidosis increases transcription of lncRNA from the IGS region located 28 kb downstream of the pre-rRNA transcription start site. The transcripts are likely ~300 nt long and are a structural determinant of A-bodies. The authors also found that heat shock induces the expression of two other specific IGS transcripts: IGS RNA 16 and IGS RNA 22 from regions located 16 kb and 22 kb downstream of the pre-rRNA transcription start site, respectively [49] (Figure 3C). IGS 16 RNA, IGS 22 RNA, and IGS 28 RNA are synthesized by pol I from a DR-rich region [TC]n or [GA]n. It is currently believed that, since these transcripts contain low-complexity repeats, they lack secondary structure. This hypothesis remains to be confirmed or refuted experimentally. Mature IGS transcripts sequester proteins to their expression sites on the rDNA cassette. These bound proteins are detected on the rDNA by ChIP, suggesting that they directly interact with chromatin [48]. How the interaction of IGS RNA, rDNA, and proteins occurs is unclear but may involve triplex or R-loop formation. R-loops are three-stranded nucleic acid structures composed of an RNA–DNA hybrid and a displaced strand of DNA. Unlike the R-loop, which involves canonical Watson–Crick base pairing between RNA and DNA, triplex formation utilizes Hoogsteen base pairing between the lncRNA and the major groove of dsDNA [117]. Two functions of A-bodies are currently known: protein sequestration and storage and local nuclear synthesis [118]. It is likely that rIGS RNAs, like pRNA and PAPAS, mediate the suppression of rDNA during the early stages of A-body formation and thus contribute to a decrease in ribosome biogenesis.

## 3. Conclusions

The unique dynamic structure of the nucleolus, formed by phase separation, determines the critical role of this organelle in cellular adaptation. The formation of various nucleolar structures is a signal and a consequence of profound metabolic changes. The mechanisms that cause these reactions are yet to be determined, but it is certain that lncRNA molecules, long considered non-functional, actively participate in this process. lncRNAs are present at low levels or are almost absent in cells under normal conditions. Accumulation of nucleolar lncRNAs is observed in response to various stresses (IGSRNA and PAPAS activation) and unusual cellular states, such as rest (PAPAS induction). However, the mechanisms providing stimulus-specific activation of these transcripts, in most cases, remain unknown. The stress-specificity of the nucleolar body composition also remains a mystery. Investigation of the mechanism of RNA–protein interactions in these structures, as well as the processes that promote aggregation and amyloid formation in the nucleolus, may shed light on the cellular and molecular pathways of pathological nuclear aggregation.
